# Effect of thermal aging on the radiopacity of universal composite resins at two thickness levels

**DOI:** 10.1007/s00784-026-06935-3

**Published:** 2026-05-25

**Authors:** Seyit Bilal Ozdemir, Busra Ozdemir

**Affiliations:** Faculty of Dentistry Department of Restorative Dentistry, Giresun University, Goksuz Street & Number 15/17, Giresun, 28200 Turkey

**Keywords:** Composite resin, Radiopacity, Radiography, Thermocycling

## Abstract

**Objectives:**

The aim of this study was to investigate the effects of material type, specimen thickness, and thermocycling on the radiopacity of universal composite resins using equivalent aluminum thickness (mm Al).

**Materials and methods:**

Four universal paste-type (Filtek Z250 Universal Restorative; FZ250, Omnichroma; OC, Vittra APS Unique; VU, and G-aenial Universal A’Chord; GU) and three universal flowable composite resins (Omnichroma Flow; OCF, Vittra APS Unique Flow; VUF, and G-aenial Universal Flo; GUF) were tested. Disk-shaped specimens (12 mm diameter) were prepared at 1 mm and 2 mm thicknesses (*n* = 10 per group). Radiographic images were obtained using a digital X-ray system together with an aluminum step wedge and enamel–dentin references. Mean gray values (MGV) were measured using ImageJ software and converted to equivalent mm Al. Specimens were subjected to 10,000 thermocycles (5–55 °C). Data were analyzed using three-way ANOVA (*p* < 0.05).

**Results:**

Radiopacity differed significantly among the tested materials (*p* < 0.001). FZ250 and VU exhibited the highest mm Al values at both thickness levels. Specimen thickness significantly affected radiopacity, with 2 mm specimens showing higher values than 1 mm specimens across all materials (*p* < 0.001). After thermocycling, statistically significant decreases were observed in the 2 mm specimens of the VU and VUF groups (*p* < 0.001). All materials exhibited radiopacity values equal to or greater than 1 mm Al at 1 mm thickness.

**Conclusion:**

All tested universal composite resins met the minimum ISO 4049 radiopacity requirement. Radiopacity was influenced by material type and specimen thickness. Thermocycling caused a decrease in radiopacity values in some composite resin groups.

**Clinical relevance:**

Thermocycling may affect radiopacity depending on material type and specimen thickness and should be considered by clinicians during radiographic evaluation of composite restorations.

## Introduction

Radiographic evaluation is a fundamental diagnostic method for monitoring the clinical performance of restorative materials and detecting secondary caries. Accurate differentiation of the restoration–tooth interface and assessment of marginal adaptation are critically dependent on radiographic analysis [1–3]. In this context, the presence of adequate and balanced radiopacity in restorative materials is essential to prevent misinterpretation of secondary caries and the unnecessary replacement of restorations [4, 5]. Insufficient radiopacity may render restorations indistinguishable from dentin, whereas excessively high radiopacity may obscure marginal defects and lead to inaccurate clinical decisions [3].

International standards recommend evaluating the radiopacity of restorative materials by comparison with an aluminum reference. According to ISO 4049, the radiopacity of a 1-mm-thick composite resin should be at least equivalent to that of the same thickness of pure aluminum [6–8]. Dentin has been reported to exhibit radiopacity values comparable to aluminum, whereas enamel demonstrates approximately twice the radiopacity of aluminum [7, 8]. Therefore, the use of an aluminum step wedge in conjunction with enamel–dentin reference structures in digital radiographic systems is widely accepted for radiopacity assessment [9–11]. In digital radiographic analysis, mean gray value (MGV) represents a directly measured parameter, whereas equivalent aluminum thickness (mm Al) is a standardized value derived from MGV measurements using an aluminum step wedge–based interpolation method, allowing a complementary evaluation of radiopacity.

Restorative materials are continuously exposed to temperature fluctuations, moisture, and water absorption in the oral environment. Thermal aging is a commonly used laboratory method to simulate these conditions and may influence the physical and chemical properties of composite resins [12, 13]. Thermocycling has been developed to reproduce, within a shorter time and under standardized conditions, the physical effects associated with long-term clinical service. According to ISO standards, thermocycling is performed between 5 °C and 55 °C, and 10,000 cycles have been reported to correspond approximately to one year of in vivo use [14–16]. Water absorption and degradation at the filler–matrix interface have been suggested to result in changes in radiopacity over time [13, 17].

Numerous studies have evaluated the radiopacity of various restorative materials [18–21]. The radiopacity of composite resins varies depending on the polymer matrix composition as well as the type, size, and concentration of filler particles containing high atomic number elements such as barium, strontium, zirconium, or ytterbium. In addition, material thickness is considered a determining factor influencing radiopacity. An increase in thickness has been associated with higher radiopacity values, whereas insufficient radiopacity in thin restorations may compromise radiographic interpretation [22, 23]. Flowable (injectable) composite resins have gained increasing clinical popularity due to their ease of application and compatibility with minimally invasive techniques [24, 25]. Flowable composite resins, similar to conventional composites, are increasingly used in clinical practice, highlighting the importance of evaluating their radiographic behavior under different conditions. However, studies simultaneously evaluating the combined effects of material type, specimen thickness, and thermocycling on the radiopacity of universal composite resins remain limited. It has been reported that restorative materials of different thicknesses may respond differently to artificial aging procedures such as thermocycling [13]. Since radiopacity is influenced by material composition, filler characteristics, and specimen thickness, evaluating these factors together is important for understanding the radiographic behavior of restorative materials over time. Therefore, differences in radiopacity levels may influence clinicians’ selection of restorative materials [17, 26]. Accordingly, the present study evaluated the radiopacity of different universal composite resins at two thickness levels before and after thermocycling. The following null hypotheses were tested:

1. Composite material type has no statistically significant effect on radiopacity.
2. Specimen thickness (1 mm vs. 2 mm) has no statistically significant effect on radiopacity.
3. Thermocycling has no statistically significant effect on radiopacity.

## Materials and methods

This study received ethical approval from the Giresun University Medical Research Ethics Committee (2025/335).

Four commercially available universal paste-type composite resins (Filtek Z250 Universal Restorative; FZ250, Omnichroma; OC, Vittra APS Unique; VU, and G-aenial Universal A’Chord; GU) and three universal flowable composite resins (Omnichroma Flow; OCF, Vittra APS Unique Flow; VUF, and G-aenial Universal Flo; GUF) were used in this study. The compositions, filler contents, and other relevant characteristics of the tested composite resins are presented in Table 1. These materials were selected as they represent commonly used universal composite resins with varying filler compositions and radiopaque elements.

**Table 1:** Composition of the tested composite resins

Composite	Manufacturer	Category	Resin matrix	Filler type	Filler content (wt% / vol%)	Clinical Application Protocol
Omnichroma (OC)	Tokuyama, Japan	paste-type composite resin	UDMA, TEGDMA	Silica and zirconium supra-nano spherical filler (260 nm)	79 / 68	No shade selection; incremental layering; light curing for 20 s.
Omnichroma Flow (OCF)	Tokuyama, Japan	Flowable composite resin	UDMA, TEGDMA	Uniform-sized supra-nano spherical filler (SiO₂–ZrO₂, 260 nm); round-shaped composite filler	71 / 57	No shade selection; incremental layering; light curing for 20 s.
Vittra APS Unique (VU)	FGM, Brazil	paste-type composite resin	Methacrylate monomer mixture, photoinitiator system (APS), co-initiators, stabilizers, silane	Boron-aluminum-silicate glass (200 nm)	72–80 / 52–60	No shade selection; incremental layering; light curing for 20 s.
Vittra APS Unique Flow (VUF)	FGM, Brazil	Flowable composite resin	Methacrylate monomer mixture, photoinitiator system (APS), co-initiators, stabilizers, silane	Barium-aluminum-borosilicate glass and SiO₂ (1.7–1.9 μm)	58–62 / 56–60	No shade selection; incremental layering; light curing for 20 s.
G-aenial Universal A’Chord	GC, Japan	paste-type composite resin	Bis-MPEPP, UDMA	300 nm barium glass filler, 16 nm fumed silica, organic filler	82 / 65	A2 shade; incremental layering; light curing for 20 s
G-aenial Universal Flo (GUF)	GC, Japan	Flowable composite resin	UDMA, Bis-MEPP, TEGDMA	SiO₂ (16 nm), strontium glass (200 nm)	69 / 50	A2 shade; incremental layering; light curing for 20 s
Filtek Z250 Universal Restorative (FZ250)	3 M, USA	paste-type composite resin	Bis-GMA, UDMA, Bis-EMA, TEGDMA, PEGDMA	Silica particles (20 nm) and zirconia/silica particles	82 / 68	A2 shade; incremental layering; light curing for 20 s

Filler content values were reported according to the manufacturers’

An a priori power analysis was performed (α = 0.05, power = 0.80). Based on an effect size of 0.50 derived from previous studies [20], at least 10 specimens were required for each material–thickness group. In this study, seven different composite resin materials were prepared in disk form. For each material, specimens were fabricated at two different thickness levels (1 mm and 2 mm). Ten specimens (*n* = 10) were prepared for each material–thickness group, resulting in a total of 140 disk specimens. Specimens were fabricated using a silicone mold with a diameter of 12 mm and standardized thicknesses of 1 mm and 2 mm. An aluminum step wedge was used as a reference for radiopacity measurements, and a recently extracted human third molar was included as a comparative reference.

### Specimen preparation

Disk-shaped specimens were fabricated using circular silicone molds with a diameter of 12 mm and thicknesses of 1 mm and 2 mm. All composite resins were placed into the molds in a single increment. After complete filling of the molds, a Mylar strip was applied to the surface to minimize the oxygen inhibition layer and to obtain a smooth surface. A glass slide was then positioned over the strip to remove excess material and ensure surface flatness. Polymerization was performed using an LED light-curing unit (Woodpecker Medical Instrument Co., Guilin, China) operating at an intensity of 1000 mW/cm². The curing procedure was carried out according to the manufacturers’ recommendations, and the clinical application protocol for each composite resin is presented in Table 1. The output intensity of the curing unit was verified using a radiometer prior to each use to ensure standardization across all groups. Following polymerization, all specimens were sequentially finished with 800- and 1200-grit silicon carbide abrasive papers to eliminate surface irregularities. The final thickness of each specimen was verified using a digital caliper (Absolute Digimatic, Mitutoyo Corp., Kawasaki, Japan). All prepared specimens were stored in distilled water at 37 °C under dark conditions for 24 h prior to measurement. For the enamel and dentin reference specimens, a recently extracted human third molar free of caries, cracks, or structural defects was used.

### Radiographic analysis

**Fig. 1:**
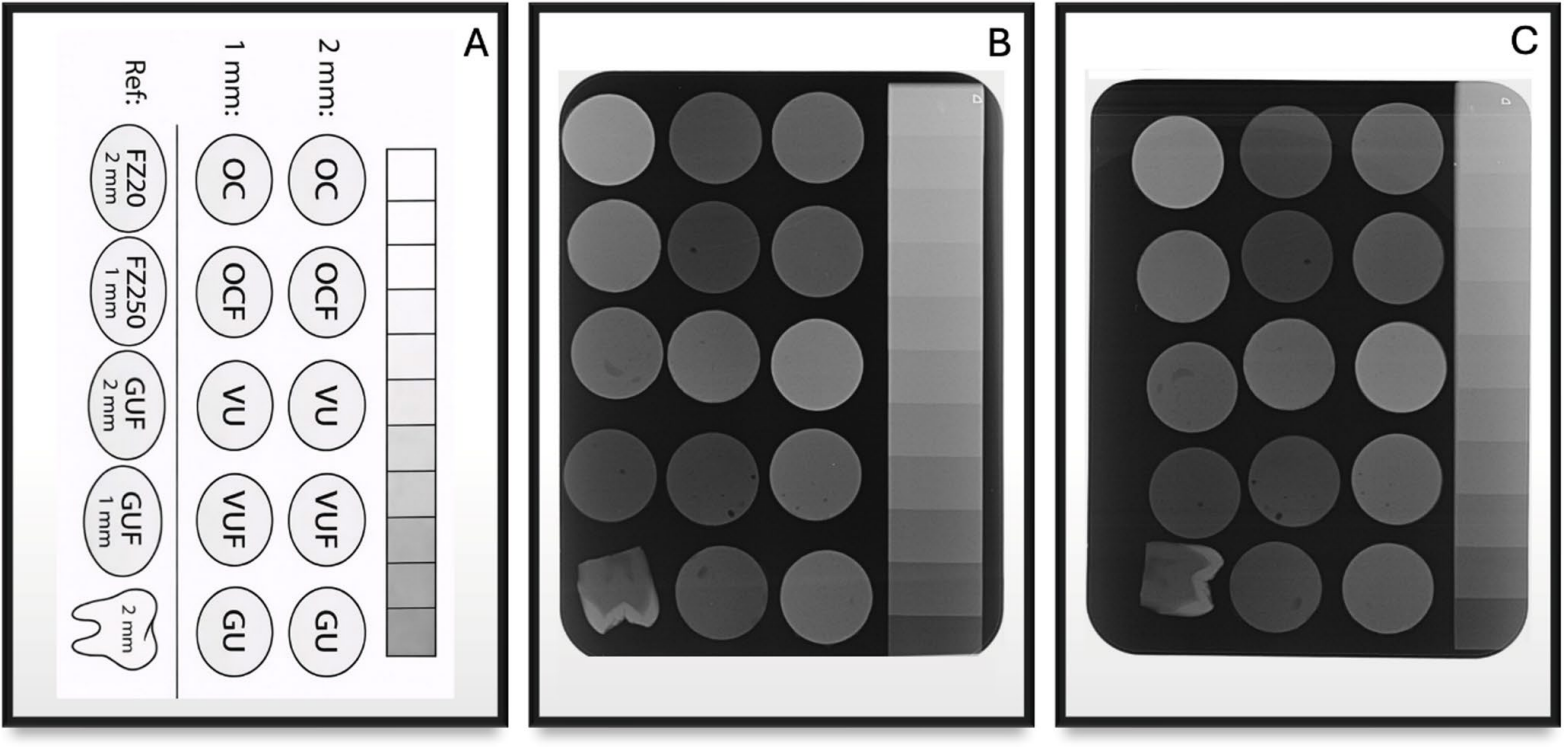
(**A**) Schematic representation of specimen identification and positioning. (**B**) Radiographic image before thermocycling. (**C**) Radiographic image after thermocycling. All specimens were imaged together with an aluminum step wedge and a 2-mm enamel–dentin reference section

Digital radiographic images were obtained using a BEST-X-DC dental X-ray unit (New Life Radiology S.R.L., Italy). For each composite resin, 1-mm and 2-mm specimens were positioned together at the center of a size 4 phosphor storage plate (5.7 × 7.5 cm; Dürr Dental, Bietigheim-Bissingen, Germany), along with an aluminum step wedge and a 2-mm enamel–dentin section (Fig. 1). A custom-made aluminum step wedge (11-step, 99.5% purity) was used as a radiographic reference. The step configuration was prepared according to commonly accepted radiopacity standards used for calibration in dental radiographic studies [10]. All radiographs were acquired using standardized exposure parameters (60 kV, 7 mA, 0.16 s) with a fixed source-to-object distance of 30 cm. After exposure, the phosphor plates were scanned using a dedicated phosphor plate scanner (VistaScan Mini Plus, Dürr Dental, Bietigheim-Bissingen, Germany) to obtain digital images. Radiopacity measurements were performed on the digital images by analyzing the aluminum step wedge, composite specimens, and enamel–dentin regions. For each structure, three separate areas of 1 mm² (10 × 10 pixels) were selected, and mean gray values (MGV) were recorded using image analysis software. The final MGV for each specimen was calculated as the average of the three measurements.

Fourteen experimental groups were defined according to composite material (*n* = 7) and specimen thickness (1 mm and 2 mm) (Fig. 1A). Each group contained ten specimens (*n* = 10). During radiographic acquisition, one specimen from each group was placed on the same phosphor plate; therefore, ten different radiographic images were obtained for baseline measurements (Fig. 1B). The same imaging configuration was repeated after thermocycling, resulting in a total of twenty radiographic images (Fig. 1C). Radiopacity measurements were performed individually for each specimen; therefore, each specimen was considered the observational unit for statistical analysis. The MGV values obtained from each measurement were converted into equivalent aluminum thickness (mm Al) using the method described by Lachowski et al. [27]

According to this approach, the radiopacity of each test material was calculated by linear interpolation between the two adjacent aluminum steps whose MGV values were immediately above and below the MGV of the tested specimen. The following equation was applied:

$$\mathrm{mm}\;\mathrm{Al}\;=t_{low}\;+\frac{MGV_{sample}-MGV_{low}}{MGV_{high}-MGV_{low}}\times\;(t_{high}-t_{low})$$

where MGV_sample is the mean gray value of the tested specimen; MGV_low and MGV_high are the MGV values of the aluminum steps with values immediately lower and higher than that of the specimen, respectively; and t_low and t_high represent the known aluminum thicknesses (mm Al) of these adjacent steps. The value t_low defines the lower bound of the interpolation interval, while the fractional term determines the relative position of the specimen’s MGV between these two steps.

### Thermocycling procedure

The prepared specimens were subjected to artificial aging using a thermocycling device (Thermocycler SD Mechatronic, Feldkirchen-Westerham, Germany). The thermocycling process was performed in water baths maintained at 5 °C and 55 °C (± 1 °C) for a total of 10,000 cycles. The specimens were placed in the sample holder of the device and alternately immersed in cold and hot water baths.

The dwell time in each bath was set at 30 s, with a transfer time of 10 s between baths. Accordingly, the specimens were immersed for 30 s in the cold bath followed by 30 s in the hot bath. This sequence was considered one thermal cycle, and the procedure was repeated 10,000 times to complete the aging protocol [28].

Following completion of the thermocycling procedure, digital radiographic images of all specimens were obtained using the same X-ray device and identical exposure parameters as those used for the baseline measurements. MGV values were recalculated from the newly acquired radiographs using the same analysis protocol applied at baseline.

### Statistical analysis

All statistical analyses were performed using IBM SPSS Statistics for Windows, Version 27.0 (IBM Corp., Armonk, NY, USA). The normality of the data was assessed using the Shapiro–Wilk test, and all groups were found to follow a normal distribution (*p* > 0.05). A three-way analysis of variance (ANOVA) was performed to evaluate the effects of material type, specimen thickness, and thermocycling on radiopacity. Interaction effects among the factors were also analyzed. Partial eta-squared (η²) values were calculated to determine effect sizes, and 95% confidence intervals (CI) were reported. The level of statistical significance was set at *p* < 0.05 for all analyses.

## Results

**Fig. 2:**
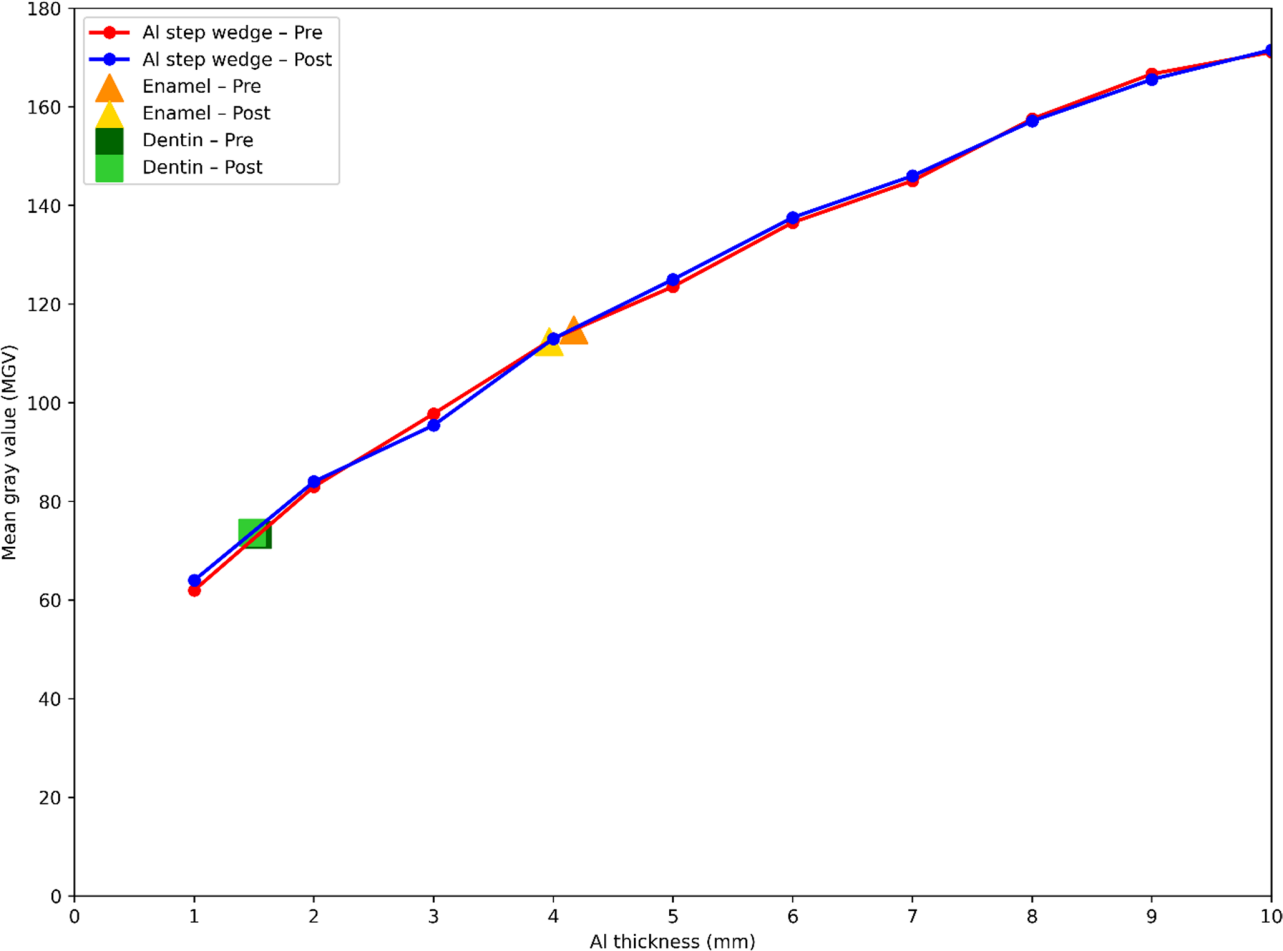
Aluminum step wedge curve and MGV distribution of enamel and dentin references before and after thermocycling; demonstrating the consistency of the radiographic measurements

The MGV values obtained from the aluminum step wedge and enamel–dentin reference structures remained within similar ranges in the radiographic images acquired before and after thermocycling. These reference measurements were used to verify the consistency of the radiographic imaging conditions and to minimize potential variability associated with the imaging system (Fig. 2). None of the tested composite resins exhibited radiopacity values below 1 mm Al at 1 mm thickness, either before or after thermocycling. At 2 mm thickness, all tested composite resins exhibited mm Al values higher than the dentin reference.

Detailed results of the three-way ANOVA are presented in Table 2. Significant main effects were found for material type, specimen thickness, and thermocycling (*p* < 0.001).

**Table 2:** Three-way ANOVA results for radiopacity according to material type, thickness, and thermocycling

Effect	*p*-value	Partial η²	95% CI
Material	< 0.001	0.982	0.978–0.986
Thickness	< 0.001	0.969	0.963–0.974
Thermocycling	< 0.001	0.597	0.522–0.661
Material × Thickness	< 0.001	0.703	0.641–0.752
Material × Thermocycling	< 0.001	0.430	0.351–0.496
Thickness × Thermocycling	0.018	0.062	0.012–0.118
Material × Thickness × Thermocycling	0.003	0.075	0.010–0.124

Partial η² values represent effect sizes, CI: confidence interval

### Inter-material comparisons

Statistically significant differences in mm Al values were observed among the composite resins at both 1 mm and 2 mm thickness levels. At both thickness levels the highest mm Al values were recorded in the FZ250 group. When composite resins at 1 mm thickness were compared before and after thermocycling, universal flowable composite resins consistently exhibited lower mm Al values than paste-type composite resins (Table 3).

**Table 3:** Equivalent mm Al values of the tested composite resins before and after thermocycling at two thickness levels

Material	Before thermocycling		After thermocycling	
	1 mm (Mean ± SD)	2 mm (Mean ± SD)	1 mm (Mean ± SD)	2 mm (Mean ± SD)
OC	1.49 ± 0.06 ^aCα^	3.03 ± 0.08^aCβ^	1.32 ± 0.05 ^aCα^	3.03 ± 0.18 ^aCβ^
OCF	1.00 ± 0.00 ^aDα^	2.05 ± 0.24^aDβ^	1.00 ± 0.00 ^aDα^	1.75 ± 0.04^aEβ^
VU	2.77 ± 0.17 ^aBα^	5.21 ± 0.10^aAβ^	2.52 ± 0.08 ^aBα^	4.25 ± 0.20^bBβ^
VUF	1.18 ± 0.13 ^aDα^	3.04 ± 0.34^aCβ^	1.00 ± 0.00 ^aDα^	1.91 ± 0.04^bEβ^
GU	1.50 ± 0.09 ^aCα^	2.82 ± 0.38^aCβ^	1.37 ± 0.08 ^aCα^	2.55 ± 0.11^aDβ^
GUF	1.18 ± 0.04 ^aDα^	2.21 ± 0.28 ^aDβ^	1.00 ± 0.00 ^aDα^	1.99 ± 0.07^aEβ^
FZ250	3.28 ± 0.29 ^aAα^	5.40 ± 0.10 ^aAβ^	3.25 ± 0.08 ^aAα^	5.16 ± 0.08^aAβ^
Enamel	-	3.58 ± 0.12^B^	-	3.58 ± 0.12^C^
Dentin	-	1.52 ± 0.06^E^	-	1.52 ± 0.06^F^

Different uppercase letters within the same column indicate statistically significant differences among materials (*p* < 0.001)

Different lowercase letters within the same row indicate statistically significant differences between thermocycling conditions within the same material at the same thickness level (*p* < 0.05)

Different symbols (α, β) within the same row and thermocycling condition indicate statistically significant differences between thickness levels within the same material (*p* < 0.001)

### Effect of thickness

Across all composite resin groups, both before and after thermocycling, specimens with a thickness of 2 mm exhibited higher mm Al values than those with a thickness of 1 mm (Table 3).

### Effect of thermocycling

**Fig. 3:**
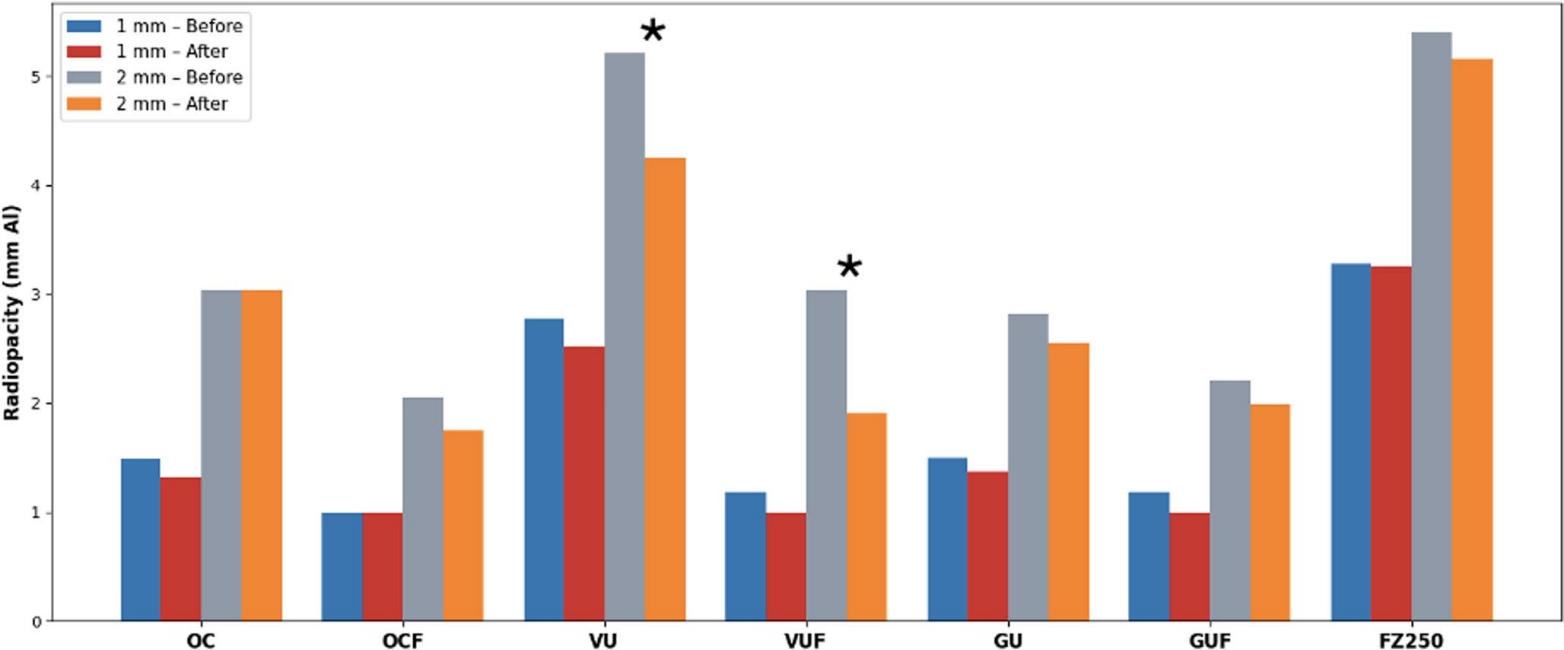
Radiopacity values (mm Al) of composite resins at 1 mm and 2 mm thickness before and after thermocycling. Asterisks (*) indicate statistically significant differences after thermocycling, observed only in the VU and VUF groups at 2 mm thickness (*p* < 0.001)

As shown in Fig. 3, a decrease in mm Al values was observed in some composite resins after thermocycling. In the 1 mm specimens, no statistically significant differences were found between the before and after thermocycling mm Al values (*p* > 0.05). In the 2 mm specimens, the VU and VUF groups demonstrated a statistically significant decrease in mm Al after thermocycling (Table 3). In contrast, the 2 mm specimens of the OCF group showed a statistically significant decrease in MGV values after thermocycling compared with baseline values (Table 4). These findings indicate that the effect of thermocycling was material-dependent.

**Table 4:** Mean gray value (MGV) measurements of the tested composite resins before and after thermocycling at two thickness levels

Material	Before thermocycling		After thermocycling	
	1 mm (Mean ± SD)	2 mm (Mean ± SD)	1 mm (Mean ± SD)	2 mm (Mean ± SD)
OC	72.3 ± 1.3 ^C^	98.3 ± 1.3^aC^	70.4 ± 1.0 ^C^	96.3 ± 2.6^aC^
OCF	58.5 ± 2.4 ^E^	83.2 ± 4.4^aD^	55.0 ± 3.1^E^	78.9 ± 0.9 ^bE^
VU	94.4 ± 2.5^B^	126.3 ± 1.3^aA^	89.9 ± 0.9^B^	116.0 ± 2.4^bB^
VUF	65.5 ± 3.1^D^	98.4 ± 5.1^aC^	51.4 ± 2.6^F^	82.1 ± 0.9^bE^
GU	72.4 ± 2.0^C^	95.1 ± 5.6^aC^	71.4 ± 1.6^C^	90.3 ± 1.3^aD^
GUF	65.8 ± 0.8^D^	86.6 ± 3.9^aD^	60.1 ± 0.9^D^	83.5 ± 1.0^aE^
FZ250	101.5 ± 4.5^A^	128.7 ± 1.3^aA^	100.0 ± 1.5^A^	127.0 ± 1.1^aA^
Enamel	-	106.0 ± 1.3^B^	-	106.0 ± 1.3^C^
Dentin	-	73.2 ± 1.6^E^	-	73.2 ± 1.6^F^

Different uppercase letters within the same column indicate statistically significant differences among materials (*p* < 0.001)

Different lowercase letters within the same row indicate statistically significant differences between thermocycling conditions within the same material at 2 mm thickness

## Discussion

In the present study, the radiopacity of universal composite resins prepared at two different thickness levels was evaluated before and after thermocycling using mm Al measurements. The findings demonstrated that composite material type had a significant influence on radiopacity, and the highest radiopacity values at both thickness levels were recorded in the FZ250 group. Therefore, the first null hypothesis, which stated that material type would not affect radiopacity, was rejected. In addition, since 2 mm specimens exhibited higher radiopacity values than 1 mm specimens across all material groups, the second null hypothesis was rejected. Following thermocycling, a statistically significant decrease in radiopacity was observed only in the 2 mm specimens of VU and VUF. Accordingly, the third null hypothesis was partially rejected.

Evaluating radiopacity not only in terms of mm Al but also using MGV enhanced the sensitivity of the measurements. In some 1 mm specimens, radiopacity values clustered at the lower detection limit of the aluminum step wedge, resulting in measurements being recorded as 1.00 mm Al. This may have limited the ability to detect subtle changes in radiopacity after thermocycling when expressed in mm Al. Therefore, additional analysis based on MGV allowed a more sensitive evaluation of potential changes, particularly within the lower radiopacity range. Previous studies have reported that MGV analysis in digital radiography can be performed using ImageJ software and standardized through the use of an aluminum step wedge [18, 21, 24, 27]. In the present study, the close agreement between MGV results and mm Al values supports the consistency of the measurement and conversion methods applied. Furthermore, consistent MGV values obtained from the aluminum step wedge and enamel–dentin reference structures across different radiographic acquisitions support the stability of the imaging conditions. This finding indicates that the observed changes in composite resins reflect material-related alterations rather than measurement variability. Consequently, the radiopacity of the composite resins could be reliably compared with clinically relevant reference tissues.

One of the principal findings of the present study was that specimen thickness had a significant influence on radiopacity. The mm Al analysis demonstrated that, across all composite resin groups, 2 mm specimens exhibited statistically significantly higher radiopacity values compared with 1 mm specimens. This finding can be explained by increased X-ray absorption associated with greater material thickness and is consistent with previous studies [13, 29]. Furthermore, this result aligns with the fundamental principles of X-ray attenuation and parallels the well-established behavior of aluminum step wedges, in which radiopacity increases proportionally with thickness [10, 30]. In addition, a greater amount of material along the X-ray path may contribute to increased photon absorption, resulting in higher radiopacity values in thicker specimens. According to ISO 4049, the radiopacity of a 1-mm-thick composite resin should not be lower than that of an equivalent thickness of aluminum [12]. In the present study, all tested materials met this minimum requirement both before and after thermocycling. From a clinical perspective, the higher radiopacity observed in thicker restorations may facilitate radiographic differentiation from adjacent tooth structures and support the detection of marginal discrepancies or secondary caries. Radiopacity is a critical property for the radiographic evaluation of restorative materials, particularly for the detection of secondary caries, assessment of marginal adaptation, and accurate identification of restoration–tooth boundaries. It has been reported that restorative materials should exhibit radiopacity higher than that of enamel to enable clear differentiation between restorations and recurrent caries [21].

In the present study, the FZ250 and VU groups exhibited radiopacity values exceeding those of enamel at 2 mm thickness both before and after thermocycling. The higher radiopacity values observed may be associated with the relatively higher filler loading of FZ250 and the presence of glass-based radiopaque filler systems in VU [17, 31]. In FZ250, this effect may also be related to the presence of high atomic number elements within the filler system, as reported in the literature [18]. Previous studies have reported that radiopacity is directly related to filler particle content and the presence of high atomic number elements such as barium, strontium, zirconium, ytterbium, and tungsten [6, 18, 24]. Similar investigations have emphasized that the type and distribution of these elements within the material significantly influence radiopacity values expressed in mm Al [18, 24]. Dionysopoulos et al. reported that composite resins may exhibit low radiopacity despite high filler content if high atomic number elements are present in limited amounts within the filler phase [6]. In this context, the relatively lower radiopacity observed for OCF, particularly at 1 mm thickness, may reflect differences in filler composition and volumetric filler content rather than filler loading alone. In contrast, the lower radiopacity observed in some flowable composite resins evaluated in the present study (VUF and GUF) may be associated with lower filler content and a higher resin matrix proportion compared with the tested paste-type materials [29, 31]. Similar trends have been reported in previous studies, which suggested that certain flowable composites may present lower radiopacity values than conventional composite resins due to differences in filler composition and matrix proportion [6, 17, 32]. From a clinical perspective, these differences in radiopacity may influence the radiographic detectability of restorations and their differentiation from surrounding tooth structures.

The 10,000-cycle thermocycling protocol applied in this study is widely accepted in the literature as simulating approximately one year of intraoral use [14–16, 28]. Therefore, the findings provide insight into a laboratory aging model designed to simulate clinical service conditions. When the effect of thermocycling was evaluated, a decreasing trend in radiopacity values was observed in some composite resin groups. According to the statistical analysis, this change was significant in the VUF and VU groups, whereas in the OCF group statistical significance was detected only in the MGV analysis. Previous studies have associated similar findings with factors such as water sorption and microstructural changes at the resin matrix–filler interface, which may influence X-ray attenuation. However, these mechanisms were not directly investigated in the present study and should therefore be interpreted as plausible explanations rather than confirmed mechanisms [33–36]. The filler content, particle size, and filler type of composite resins are known to influence water sorption and solubility [37]. Materials containing more hydrophilic components generally exhibit higher water sorption. Monomers such as TEGDMA, Bis-GMA, and UDMA contain polar functional groups that increase hydrophilicity and affinity for water [38]. In the present study, although the exact monomer composition of VU and VUF was not fully disclosed by the manufacturer, these materials may contain one or more hydrophilic monomer components, which may increase water affinity and contribute to the decrease in mm Al and MGV values observed after aging. In addition, VUF exhibited the lowest filler content among the tested materials, which may increase water sorption and contribute to the observed decrease in radiopacity. Although VU has a relatively higher filler loading, it contains smaller filler particles (approximately 200 nm). Smaller filler particles increase the filler–matrix interfacial area, which may facilitate water penetration and promote hydrolysis of silane coupling agents, potentially leading to microstructural changes and a decrease in radiopacity after aging. Furthermore, the type and proportion of radiopaque glass fillers used in composite resins may also influence radiopacity values. Glass fillers containing elements such as barium, strontium, or zirconium are commonly incorporated into restorative materials to enhance X-ray attenuation [39]. Therefore, differences in the composition and concentration of these radiopaque glass fillers may contribute to the variations in mm Al and MGV values observed after thermocycling. Composite resins with a higher resin matrix content have been suggested to be more susceptible to thermal stress associated with water sorption. In the present study, although a numerical decrease in radiopacity values was observed in most universal composite resins after thermocycling, statistically significant changes were limited to a small number of materials. These findings are consistent with the study by Çölkesen et al., which reported that radiopacity alterations following thermocycling may vary depending on the material [13]. From a clinical perspective, such material-dependent changes in radiopacity should be considered when interpreting radiographic images.

The findings of this study should be interpreted in light of certain limitations. This study was conducted under in vitro conditions and may not fully replicate all biological and mechanical factors present in the oral environment. In addition, radiopacity measurements were performed using a single digital radiographic system, and different imaging systems may yield variable results. Furthermore, microstructural changes associated with aging were not confirmed by SEM or elemental analysis. Future studies incorporating different imaging techniques and microstructural analyses are recommended.

## Conclusion

Within the limitations of this in vitro study, the radiopacity of the tested universal composite resins was influenced by material type. FZ250 and VU exhibited the highest mm Al values at both thickness levels.

Radiopacity was also affected by specimen thickness, with all tested composite resins showing higher mm Al values at 2 mm compared with 1 mm. All composite resins exhibited radiopacity values higher than dentin at 2 mm thickness and met the minimum ISO 4049 requirement of 1 mm Al at 1 mm thickness.

Thermocycling resulted in material- and thickness-dependent changes in radiopacity. At 2 mm thickness, statistically significant decreases in both mm Al and MGV values were observed in the VU and VUF groups, whereas in the OCF group, the decrease was significant only in the MGV analysis.

These findings indicate that the effect of thermocycling on radiopacity depends on material type and specimen thickness.

## Data Availability

The dataset generated and/or analyzed during thecurrent study are available from the corresponding author on reasonablerequest.
